# Critical Considerations for Expanding Indications for Nonvascularized Rectus Fascia Transplantation: Clarifying Definitions, Techniques, and Immunogenicity

**DOI:** 10.1097/TXD.0000000000001739

**Published:** 2024-11-15

**Authors:** Laurens J. Ceulemans, Ewout Muylle, Nele Van De Winkel, Gabriel Gondolesi

**Affiliations:** 1 Leuven Intestinal Failure and Transplantation (LIFT) Center, University Hospitals Leuven, Leuven, Belgium.; 2 Department of Chronic Diseases and Metabolism, Laboratory of Respiratory Diseases and Thoracic Surgery (BREATHE), KU Leuven, Leuven, Belgium.; 3 Department of Thoracic Surgery, University Hospitals Leuven, Leuven, Belgium.; 4 Department of Abdominal Surgery, University Hospitals Leuven, Leuven, Belgium.; 5 Department of Development and Regeneration, Unit of Urogenital, Abdominal and Plastic Surgery, KU Leuven, Leuven, Belgium.; 6 Medstar Georgetown Transplantation Institute, Washington, DC.

Nonvascularized rectus fascia (NVRF) transplantation (Tx) has become part of the surgical armamentarium in the context of abdominal wall closure following intestinal or multivisceral Tx, showing promising short- and midterm outcomes.^1^ In a study by Justo et al, excellent results were also observed in elective hernia repair post–liver Tx, as previously described.^2^ Based on our experience, we would like to emphasize several critical factors for further development of this procedure.

First, the use of clear definitions in this field is crucial. As highlighted in our recent review, it is important to distinguish between full-thickness abdominal wall vascularized composite allograft (AW-VCA) Tx and NVRF Tx.^1^ Essentially, Justo et al described a technique that was originally developed by Gondolesi et al^3^ in 2009, a method recently visualized by our group. Unfortunately, the authors did not cite this foundational work, instead presenting “their own technique” as a variation of the full-thickness AW-VCA Tx method described by Levi et al in 2003.^4^

The authors’ alternative technique involves perfusing the full-thickness AW-VCA graft with a preservation solution before isolating the rectus fascia for transplant, suggesting that perfusion improves donor fascia fibroblast survival. However, there is no substantial evidence to support this claim. Other than the perfusion, this technique closely mirrors the original NVRF method.^3^ Given the excellent clinical outcomes reported with NVRF Tx in various organ transplant recipients, including liver and kidney, we question the added value of perfusion, which introduces unnecessary complexity.^1^

Recent histological analyses from our group further support this viewpoint. We consider NVRF to be a temporary scaffold that facilitates the deposition of new fibrotic tissue from the recipient as part of a foreign-body reaction.^5^ We do not believe that donor fascia fibroblasts play a role in this process because they are unlikely to survive due to ischemic conditions. Neovascularization of the NVRF graft typically takes weeks or months since no vascular anastomoses are performed.

Although we agree with Justo et al that the clinical results are encouraging and suggest expanding the use of NVRF Tx, several key questions remain. These include concerns about the risk of infection and the immunogenicity of the fascia graft. Our recent research in rabbit and rodent models, as well as existing clinical data on the development of donor-specific antibodies, shows that NVRF is not immune inert.^1,6^ Therefore, the current study lacks a detailed analysis of allorecognition and immune response toward the NVRF graft. A comprehensive understanding of this immune signaling is crucial, particularly if NVRF Tx is to be used in immunocompetent patients, as previously proposed.^1^

We recommend regular immunological monitoring and obtaining fascia biopsies during reoperations to better understand immune signaling. Additionally, radiological monitoring should be used to detect complications such as seromas, fascia loosening, or herniation.

In conclusion, NVRF Tx has demonstrated excellent outcomes in abdominal wall closure and hernia repair, with significant potential for broader applications. Although this procedure is highly promising, further research on immunogenicity, ingrowth mechanisms, and potential complications will be essential to ensure its long-term success and wider implementation.

## ACKNOWLEDGMENTS

The authors thank the Leuven Intestinal Failure and Transplant team for the support in the clinical program of NVRF Tx and Ina Henion, Marina Gabriela Mori da Cunha, and Lieven Thorrez for their support in the preclinical model at Leuven.

## References

[1] Muylle E Van De Winkel N Hennion I. Abdominal wall closure in intestinal and multivisceral transplantation: a state-of-the-art review of vascularized abdominal wall and nonvascularized rectus fascia transplantation. Gastroenterol Clin North Am. 2024;53:265–279. 38719377 10.1016/j.gtc.2023.12.001

[2] Justo I Caso O Marcacuzco A. Hernia correction after liver transplantation using nonvascularized fascia. Transplant Direct. 2024;10:e1662–e1665. 38911273 10.1097/TXD.0000000000001662 PMC11191961

[3] Gondolesi G Selvaggi G Tzakis A. Use of the abdominal rectus fascia as a nonvascularized allograft for abdominal wall closure after liver, intestinal, and multivisceral transplantation. Transplantation. 2009;87:1884–1888. 19543069 10.1097/TP.0b013e3181a7697a

[4] Levi DM Tzakis AG Kato T. Transplantation of the abdominal wall. Lancet. 2003;361:2173–2176. 12842369 10.1016/S0140-6736(03)13769-5

[5] Muylle E Maes A De Hertogh G. Multilevel analysis of the neovascularization and integration process of a nonvascularized rectus fascia transplantation. Transplant Direct. 2024;10:e1624–e1628. 38757048 10.1097/TXD.0000000000001624 PMC11098214

[6] Van De Winkel N da Cunha MGM Dubois A. Allogeneic abdominal non-vascularized rectus fascia transplantation without immunosuppression equals syngeneic transplantation in a rabbit model at short-term follow-up. Transpl Immunol. 2024;87:102138. 39442588 10.1016/j.trim.2024.102138

